# An evidence-based digital prescription opioid safety toolkit for national dissemination: co-design and user testing

**DOI:** 10.3389/fdgth.2025.1600836

**Published:** 2025-07-11

**Authors:** Alex Waddell, Jessica L. Watterson, Dhruv Basur, Christopher Owen Prawira, Louisa Picco, Tina Lam, Patrick Olivier, Joshua Paolo Seguin, Liam Kay, Suzanne Nielsen

**Affiliations:** ^1^ Action Lab, Faculty of Information Technology, Monash University, Melbourne, VIC, Australia; ^2^ Deakin University School of Public Health and Social Development, Deakin University, Melbourne, VIC, Australia; ^3^ Monash Addiction Research Centre, Eastern Health Clinical School, Monash University, Melbourne, VIC, Australia

**Keywords:** opioids, co-design, naloxone, health literacy, implementation science, human-computer interaction, behavioural science

## Abstract

**Introduction:**

Australia has one of the highest rates of opioid prescribing and prescription opioid-related harm in the world. Although effective for pain relief, the use of prescription opioids is a leading cause of preventable morbidity and mortality. Barriers exist for consumers identifying their own risk factors, accessing naloxone (opioid overdose antidote) and overdose prevention education. This study aimed to co-design a digital Opioid Safety Toolkit for national dissemination through pharmacies to encourage three consumer opioid safety behaviours: (1) uptake of naloxone, (2) creating a safety plan, and (3) discussing their use of opioids, including any concerns with their healthcare professional.

**Methods:**

The digital Toolkit was co-designed and developed using a novel approach to digital health intervention design combining the Theoretical Domains Framework (TDF) and Double-Diamond design process. Co-design involved a series of seven iterative workshops with consumers (4) and professionals (3). Workshops focused on identifying factors influencing opioid safety behaviours, exploring design preferences, sense-checking, and ideation of the user flow. User testing was conducted with the penultimate version of the Toolkit.

**Results:**

13 consumers with lived experience of prescription opioid use and 14 professionals including prescribers, pharmacists, pain specialists, researchers and consumer advocates participated in up to three separate workshops. 15 consumers participated in user testing interviews. Analysis of workshops identified factors promoting safety behaviours including increased public awareness of naloxone, understanding personal risk (TDF domain of Knowledge); healthcare professional's role in education and consumers' experience of stigma (Social/professional role and identity); use of conversational aids to scaffold conversations, material resources and data ownership (Environment, context and resources). User testing elicited feedback pertaining to the information and resources on the website and the overall user interface and experience.

**Discussion:**

The Toolkit was co-designed with consumers and professionals to facilitate opioid safety behaviours. The Toolkit includes evidence-based information, tools for risk assessment and screening, opioid use monitoring, conversation aids, and a safety plan. The Toolkit is being disseminated nationally through Australian pharmacies following a randomized controlled trial that demonstrated the Toolkit promotes safety behaviours, is easy to use and acceptable to those with lived experience of prescription opioid use and professionals.

## 1 Introduction

Opioid use and related harms are a global public health challenge, resulting in considerable health, economic and societal impacts. One of the most substantial harms is fatal and non-fatal overdoses. While the types of opioids involved in opioid-related deaths vary across countries, in Australia, harms associated with opioids are predominantly related to prescription opioids (1). Opioid overdose deaths in Australia have increased two-fold between 2002 and 2019 (2), with the majority involving prescription opioids (1, 3). Unlike the US and Canada, Australia did not see an escalation of opioid deaths during the COVID pandemic, opioid overdose deaths declined slightly during 2020–2021 (4, 5). However, preliminary estimates for 2022 indicate an upward trend (4). Furthermore, each day, there are around 150 hospitalizations and 14 emergency department presentations involving opioids (6), highlighting the need to identify and empower those at risk of prescription opioid related harm.

Opioids are commonly prescribed to treat both acute and chronic pain, with the latter being one of the most common reasons people seek medical care (7). Opioid prescribing has increased substantially over the past three decades and Australia has one the highest rates of opioid prescribing per capita, exceeding countries such as the US (8), with approximately 3 million Australians prescribed opioids, and 1.9 million adults initiating opioids each year (9). Long-term prescription opioid use is associated with harms including dependence, morbidity, and mortality (10).

Over the past decade there has been considerable research to understand and measure opioid-related risk among people who are prescribed opioids. For example, an Australian cohort study found 40% were prescribed high opioid doses (above 90 mg oral morphine equivalent) (11), with higher opioid dose associated with higher odds of multiple physical and mental health issues, nonmedical opioid use and opioid dependence (11). Similarly, a large proportion of those prescribed opioids had increased risk of overdose due to meeting criteria for previous alcohol use disorder (one third) or taking concurrent benzodiazepines (one third) (12–14). Other overdose risk factors include concurrent respiratory conditions and taking an opioid dose above 50 mg of oral morphine equivalents (15).

Despite an estimated four out of five people who are prescribed opioids for chronic pain having at least one opioid overdose risk factor, recognition of opioid-related risk and knowledge about signs and symptoms of opioid overdose in this population is low (15, 16). There are a range of barriers to help-seeking for this population. For example, even though one in three people in a sample of people who were prescribed opioids met criteria for opioid use disorder (OUD), less than 5% had received evidence-based treatment for it, with seeking help from a health professional reported by only a small fraction of people who were worried about their own opioid use (17).

As opioid-related risk is dynamic, routine monitoring and assessment is recommended. Provision of naloxone, an opioid overdose antidote, has been shown to reduce the likelihood of later emergency presentations, and has been shown to be acceptable for people who are prescribed opioids for pain (15, 18). An Australian Government pilot program made naloxone free from participating community pharmacies, without a prescription, however it was estimated that only 2% of at-risk people who were prescribed opioids in this study received naloxone (19). The evaluation recommended that dedicated efforts were needed to upscale overdose prevention for this group. Further, self-administered screening tools to identify OUD and monitor outcomes with opioids have been developed and shown to be acceptable to people who are prescribed opioids for pain (20–23). Taken together, the foundation, or basic tools to help identify and respond to opioid-related risk among people who are prescribed opioids have been developed, and the digitization and dissemination of these tools could promote the uptake of naloxone.

Digital health interventions aim to increase reach, equality, and use of evidence-based information compared to their non-digital counterparts (24). Digital health interventions for opioid risk management include exploration of technologies to prevent, predict, detect, and respond to opioid misuse and overdose (25, 26). For example, technologies for prevention of opioid overdose have included computational methods to predict relapse and recovery factors, digitally mediated peer sponsorship, and design of drug checking test result displays to depict uncertainty (27–29). While for detection of and response to opioid overdose there is research around hypothetical and deployed web-based apps which alert volunteers to nearby potential overdoses (30, 31). However, two recent systematic reviews found a lack of studies reporting satisfaction, acceptability, willingness of consumers to engage with the digital technologies (25, 26).

Researchers have called for future work to use co-design approaches with consumers who are prescribed opioids for pain relief, especially those with chronic pain (32, 33). However, most of the research to date concerning the design of digital health interfaces has focused on those who use illicit opioids or misuse prescription opioids, rather than those prescribed opioids for pain relief. Furthermore, these studies tend to focus on the design of digital interventions at a local level (e.g., crowdsourcing opioid antidote provision in confined areas (34) rather than design for implementation at an organisational or systems level (e.g., nationwide) (35, 36).

A critical reason for failure to implement and scale digital health interventions is the lack of consideration for contextual and implementation factors across individual (behavioural), organization, and system levels during the design process. Mohr (37) highlights that technology-enabled services are often developed by those outside implementation settings, neglecting key considerations like requirements, processes, and service needs (38). Implementation science is the scientific study of what works to embed evidence-based interventions, programs and policies in real-world settings (39). Several theories, models and frameworks allow researchers and practitioners to understand contextual factors and develop strategies to influence adoption, ongoing maintenance, and scale of interventions (40). For example, determinant frameworks focus on predicting or explaining factors affecting implementation by identifying contextual barriers and facilitators thereby helping plan for solutions to problems before they arise [e.g., the Theoretical Domains Framework (TDF)] (40, 41).

Researchers and practitioners see the potential benefits in combining human-computer interaction (HCI) and implementation science (42, 43). However, most of the research to date focuses on how implementation science can benefit from HCI, rather than how HCI may benefit from implementation science. For example, promoting iterative design and evaluation in implementation planning (37) and embedding human-centered and user-centered approaches to the development of implementation strategies (42–44). Waddell et al. (46) addressed this gap by proving an approach to combining an implementation science framework (the non-adoption, abandonment, scale-up, spread, and sustainability framework) within a design process, the Double Diamond (45–47). Although providing a systematic scaffolding through which to design for contextual factors across individual, organisational and system levels, the authors call for future work to use different implementation science frameworks depending on the design problem.

This research addresses a specific gap in the literature, the lack of digital public health interventions to empower people who have been prescribed opioids for pain relief to engage in safety behaviours. As such, the aim of this study was to co-design an opioid safety Toolkit with consumers and experts for national dissemination in pharmacies. The design of the Toolkit focused on promoting three safety behaviours (having naloxone at home, creating a safety plan, and speaking to a healthcare professional about their use of opioids, including any concerns with their opioids). Specifically, the objectives of the study were to (1) digitize pre-existing validated tools to reduce opioid safety risk factors, (2) explore the barriers and facilitators to opioid safety behaviours using a novel approach to combing implementation science and design methods, (3) design an opioid safety Toolkit that the addresses the elicited barriers and facilitators, meets the needs of end-users and is implementable in the real-world.

## 2 Method

### 2.1 Study design

**Figure 1:**
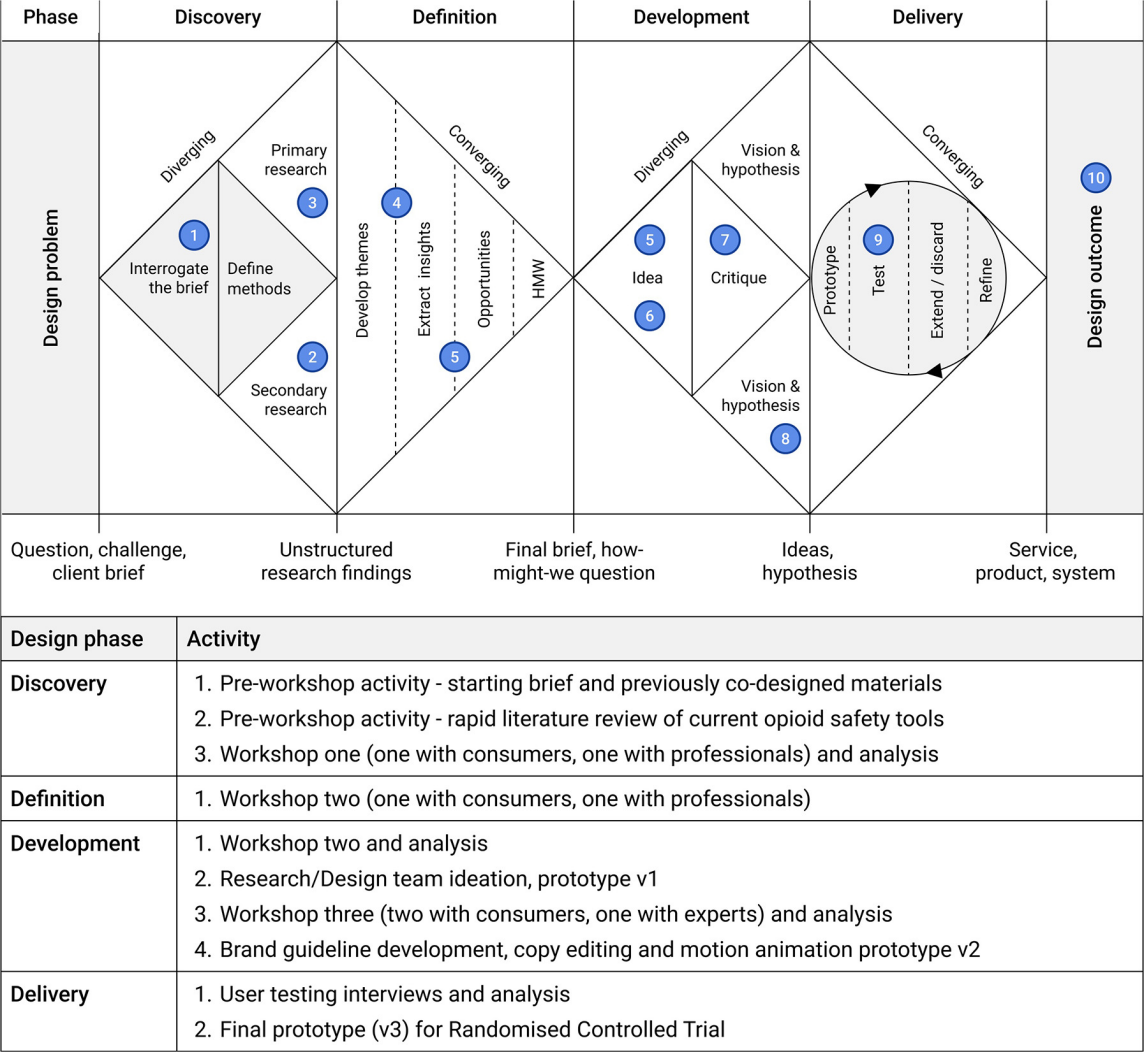
The opioid safety toolkit design process as guided by the double diamond process and relevant activities. Adapted with permission from “How to apply a design thinking, HCD, UX or any creative process from scratch” by Dan Nessler.

This study adapted a novel co-design approach that combined the Double Diamond design process alongside a behavioural and implementation science framework, the Theoretical Domains Framework (TDF) (40) (Figure 1). The Double Diamond (45) is a process framework that includes four phases from a design problem through exploration (*discovery*), synthesis (*definition*), ideation (*development*) and solution testing and refinement (*delivery*) to arrive at a design solution. Although visually represented as sequential steps, the Double Diamond can include iteration across and within the phases. As explored by Waddell et al. (46), the Double Diamond can serve as an overall process in which implementation science frameworks can be embedded (46). Given the initial focus of the Toolkit was to encourage behaviour change among prescription opioid consumers and by extension healthcare professionals, this study embedded the TDF.

The TDF is the result of the expert synthesis and simplification of behaviour change theories. The framework is used to explore and inform intervention and implementation design by eliciting data with respect to people's values, beliefs, experiences and motivations that underpin behaviour (40). The TDF consists of 14 domains and 84 component constructs that can act as barriers and facilitators to behaviours, such as knowledge, skills, environment, reinforcement, and social influences. These factors are commonly used to develop theory-informed behavioural interventions, such as public health campaigns and the uptake of new policies or procedures as it is readily mapped to the Behaviour Change Techniques Taxonomy via the Theory and Techniques Tool (48–51).

### 2.2 Participants

To maximize the real-world implementation of the Toolkit, the project team partnered with two expert organizations across the design process, representing both consumers and pharmacists. Painaustralia is a leading advocacy group working to improve the quality of life of people living with chronic pain. The Pharmaceutical Society of Australia is the peak body for Australian pharmacists. Participants were recruited using purposive and snowball sampling (52, 53). For consumers, the recruitment and sampling strategy was purposefully broad and inclusive to encourage a range of views of people who had been prescribed opioids for pain relief. Specifically, the inclusion criteria for both the co-design workshops and user testing were for people living in Australia, with lived experience of being prescribed opioids for noncancer pain relief. Exclusion criteria were people who did not have proficient English language skills to engage in the recruitment, workshops or interviews. For professionals the inclusion criteria included healthcare professionals who were responsible for prescribing or dispensing opioids for pain relief to consumers or who were members of professional or consumer advocacy groups. Exclusion criteria were professionals not practicing in Australia. Consumer participants were recruited through online advertisements via Painaustralia's LinkedIn and Facebook pages, short term consumers were invited through authors' and staff at Painaustralia's consumer networks. Health professionals and stakeholders were invited through authors' and staff at the Pharmaceutical Society of Australia's professional networks and snowball recruitment via email. Consumer participants included: (i) consumers who had been using opioids for over 3 months (long-term), (ii) consumers who had been using prescription opioids for 2-weeks to 3 months, (iii) consumers who had been using opioids for less than 2 weeks (short-term) or who were carers of people who had been prescribed opioids. Professional participants included prescribers, pharmacists, researchers, or consumer or healthcare professional advocates. Healthcare professionals from rural areas, and those with expertise working with first nations people were purposively recruited.

#### 2.2.1 Ethical considerations

Ethics approval was granted by Monash University (ID: 40628). All participants provided written informed content to prior to participation. Consumers were contacted by Painaustralia staff who have a high level of content knowledge about opioid prescriptions and chronic pain and ensured through discussion over the phone that the participant was eligible and interested in participating. “Short-term” consumers were contacted by the research team via email and were available to answer any questions over the phone. Professional participants were contacted via email and a researcher was available to answer any questions via email or phone. Privacy and confidentiality of participants was maintained by removing identifying information during the transcription process. Participant codes were assigned to participants for all analysis and reporting. All participants who participated in the co-design received $100AUD (∼$65USD) gift card for their time. Participants in the user-testing received $50AUD (∼$32.50USD) gift card for their time.

### 2.3 Overview of the co-design process

The prescription Opioid Safety Toolkit (the Toolkit) was designed and developed over 9 months using co-design processes. A total of seven 2-h workshops, three with professionals and three with consumers (workshop 3 was repeated with a different cohort of consumers for a total of four workshops) were conducted. User testing interviews were later conducted to test the usability of the Toolkit. All workshops and interviews were conducted online using the video conference platform *Zoom* (version 6) and digital whiteboard *Miro*. The *Miro* whiteboard was controlled by the researchers who screen-shared the whiteboard during the co-design workshops so that all participants could see participants' responses to activities in real-time.

#### 2.3.1 Pre-workshop activities (1, 2)—discovery

A range of previously co-designed Australian resources were identified as starting materials to include in the Toolkit, alongside examples of international resources that had been developed to increase opioid safety. The local resources were developed with Australian consumers and healthcare professionals to promote opioid safety behaviours (20–23). They included the validated Routine Opioid Outcome Monitoring (ROOM) tool which -was specifically developed to screen for prescription opioid-related risks and clinical outcomes, comprising content and language salient to people prescribed opioids for chronic non-cancer pain (20, 23). In addition, a consumer-facing leaflet and educational videos were developed in consultation with pharmacists and consumers to educate consumers on the storage and use of naloxone (54). A rapid literature review of existing opioid safety resources was conducted, and these materials were used to inform activities in Workshop 1.

#### 2.3.2 Workshop One (3)—discovery

Barriers and facilitators for the safety behaviours of interest (having naloxone at home, creating a safety plan, and speaking to a healthcare professional) were identified using three design methods. Firstly, “think aloud” (55) was used to explore consumers' and professionals' reflections on available opioid safety materials. Secondly, “journey mapping” (56) visually represented consumers' experience being prescribed opioids for pain relief (Supplementary Figure S1). Thirdly, “brainstorming” explored where and how safety behaviours might be embedded in the consumer journey for maximum uptake. Workshop transcripts were analyzed using the TDF to elicit the specific barriers and facilitators to engaging in safety behaviours. Design preferences were inductively analyzed and broadly covered the preferred language and visual design style (color, typography and visual imagery).

#### 2.3.3 Workshop Two (4,5)—definition and development

This workshop sense-checked barriers and facilitators to safety behaviours, and the language and design preferences identified in Workshop One. Participants were presented with a summary of Workshop One findings, then asked if there was nuance missing, or if anything stood out as particularly important. Workshop Two also focused on developing solutions to the issues identified in Workshop One. Participants reviewed key challenges, such as the need to tailor information based on how long someone has been on a medication. They then brainstormed solutions such as constructing screening questions to enable personalized information delivery. Finally, they helped map the Toolkit's user flow, determining the content and its optimal presentation order.

#### 2.3.4 Research/design team ideation (6)—development of v1 prototype

Following Workshop Two, the research/design team took the potential screening questions and user flows developed by the participants and used them to inform the design of an early low fidelity prototype (v1) of the Toolkit on *Figma*, an online collaborative design and prototyping tool (Supplementary Figure S2). Barriers and facilitators aligned with the TDF were mapped to relevant Behaviour Change Techniques (BCTs) using the Theory and Techniques Tool (51).

#### 2.3.5 Workshop three (7)—development of v2 prototype

This phase aimed to critique the low-fidelity prototype and refine the language and design preferences of the Toolkit. Three workshops were run, each tailored to the participant group: (i) long-term consumers of opioids, (ii) professional groups, and (iii) short-term consumers of opioids and carers. The long-term consumers and professional groups, provided feedback on each page of the v1 prototype—what their initial reactions were, what they thought the page was asking them to do, and specific feedback such as whether anything was missing, whether the flow was logical, and their preferences about the user interface design. Participants were then shown examples of language that could be used in the Toolkit (e.g., about naloxone uptake) and to provide feedback and reflections on how the language made them feel, who it was coming from, and what they thought they should do next (i.e., did it elicit behaviour change).

A new group of short-term consumers of opioids and carers were included for this workshop phase. The addition of short-term consumers was in response to participants' belief that different information should be provided to short-term consumers compared to those that had medium- or long-term prescription opioid use. These participants were first oriented to the aims of the project and the previous co-design findings. They were then shown one of the existing opioid resources to check if they identified similar barriers and facilitators to safety behaviours as previous workshop participants. Finally, they were also asked to provide page by page feedback on the v1 prototype of the Toolkit.

#### 2.3.6 Development of brand guideline, copy editing, and motion animation and development of prototype v2 (8)—development

A brand guideline was developed using synthesized feedback on preferred language and visual design style (color, typography and visual imagery). The brand guidelines served as a resource to communicate the design preferences of the workshop participants, including the general look and feel, as well as the specific colors, logos, typography, and iconography. This brand guideline was used to communicate with a copy editor who was employed to refine the language of the Toolkit in line with the learnings from the workshops. The guideline was also used to communicate with a motion animation professional who was employed to update the pre-existing opioid education videos (see pre-workshop activities section) in line with the Toolkit's branding. The v1 low-fidelity prototype (i.e., a simplified representation of the Toolkit including the user-flow and basic layout) was updated to create a more refined prototype (v2) in Figma (Supplementary Figure S2, Supplementary Figure S3). The v2 prototype incorporated participant feedback and included the brand guidelines.

#### 2.3.7 User testing interviews (9)—delivery

The v2 prototype subsequently underwent user testing with participants who had not yet interacted with the Toolkit and represented a diverse range of consumer types. During the interview, participants were provided with a link to the prototype and asked to interact with the Toolkit using “think aloud” (55) to share their thoughts as they progressed through the pages. Participants were prompted to share all thoughts, no matter how small. Interviews were recorded and transcribed and researchers took notes during the session. Feedback gathered from the interview participants was then analyzed and further refinements were made to v2 prototype's information architecture (user flow) and user interface.

#### 2.3.8 V3 prototype development (10)

The engineering team developed a mobile- and desktop-responsive web application based on the prototypes, analysis and feedback from previous phases.

### 2.4 Analysis

All workshops and interviews were video and audio recorded (via Zoom) and transcribed verbatim (using Rev.com). For the workshops, qualitative analysis was based on Atkins et al. (40) recommendations for conducting TDF-informed analysis and included both inductive and deductive approaches using NVivo software. After reading workshop transcripts to ensure familiarity, three members of the research team (AW, JW, DB) individually coded a subset of workshops (approximately 10%). Deductive coding was based on direct content analysis (57) wherein data was directly coded to the TDF domains, while any remaining transcript content was inductively coded (58). Team members met to compare and discuss coding choices and reach a consensus. One researcher (AW) coded the remaining transcripts using the same approach and developed inductive themes within each TDF domain. These themes were discussed with the overall research team who interrogated and confirmed the themes. User testing interviews were inductively analyzed (58). Three researchers (AW, DB, and CP) iteratively developed codes following each interview based on positive and negative feedback, one researcher (AW) subsequently analyzed the transcripts based on the elicited codes using NVivo.

## 3 Results

A total of 27 people participated in the workshops including 13 consumers and 14 professionals (denoted as C and P, respectively) while 15 consumers participated in the user testing interviews (Table 1). Short-term consumers included those with prescriptions for either acute or chronic pain, medium and long-term consumers included those with prescriptions for chronic pain. One professional provided feedback via email only. Demographics of participants and the workshops or interviews they attended are included in the supplementary materials (Supplementary Table S1, Supplementary Table S2).

**Table 1:** Participants and their expertise.

	Number of participants^a^	
	Workshops	User testing
Consumers		
Long term prescription opioid use (more than 3 months)	10	12
Medium term prescription opioid use (2 weeks to 3 months)	0	1
Short term prescription opioid use (less than 2 weeks)	2	2
Carer	1	0
Total number of consumers	13	15
Healthcare professionals and stakeholders		
Community pharmacist	4	
Pharmacist specializing in pain management	4	
Prescriber pain specialist	2	
Academic pharmacist/researcher	2	
Stakeholders who worked for consumer or healthcare professional advocacy groups	2	
Total number of healthcare professionals	14	

^a^ Each participant is counted once in the table, some participants attended multiple co-design workshops (detailed in the supplementary materials).

### 3.1 Workshop results

Analysis of the workshops identified five TDF domains and subsequent sub-themes—Knowledge (increasing public awareness, understanding personal risk); Social/Professional Role and Identity (healthcare professionals role and responsibilities, experiences of stigma); Environment, Context and Resources (a conversational aid, material resources and data ownership); Social Support (others need to know how to use naloxone); and Beliefs about Capabilities (consumers are experts in their own experience).

#### 3.1.1 Knowledge

##### 3.1.1.1 Public awareness

Consumers felt it was crucial that the general community know about the importance of having naloxone on hand for opioid safety and its availability at pharmacies. For them, increasing public awareness should result in the people who are prescribed opioids for pain keeping naloxone in their first aid kit and knowing how to use it in case of an emergency. Importantly, their explanation of naloxone's importance included an additional message—that opioid overdose could happen to anyone, rather than only to people who were misusing or using illicit opioid substances.

“I think educating the wider community so that naloxone is not looked at as it's the druggies, that it is part of your First Aid thing and so that everyone realizes that it could just save someone in your family or one of your friends or something.” C06

Professionals said it was crucial that for safety behaviours to change, especially increasing naloxone uptake, there would need to be a public health campaign. Interestingly, all experts agreed there would need to be a campaign that reached both consumers and professionals using the same visuals and branding to ensure consistency. This finding aligned with consumer's request that experts be provided with education and training to be prepared for any potential conversations with consumers, especially around naloxone uptake. Likewise, consumers felt that consistent visuals and branding of a resource being promoted to both professionals and consumers would increase the credibility and therefore make it more likely for professionals to support them if they were to discuss the Toolkit with their own healthcare professional.

“So the primary issue with naloxone is normalizing it to such degree that people are comfortable with it being part of their life. So for both people who are using opioids and also the prescribers and dispensers and other health professionals who work in that space” P12

##### 3.1.1.2 Understanding personal risk

Consumers felt a lack of knowledge of the risks associated with opioid use was a barrier to engaging in safety behaviours. Many of the consumers thought that those with new prescriptions should have the risks explained to them, by healthcare professional or through provision of resources. Although most long-term opioid consumers in the workshops felt they knew “enough” information about the risks associated with prescription opioids, some lamented having to find out information about risks themselves often after trial and error with medications or in some cases accidental overdose (e.g., sedation and slowed breathing). Interestingly, even though most long-term opioid prescription consumers felt although they knew “enough” information about risks, they expressed that it was still helpful for them to see the information again.

“Speaking for myself, probably be helpful for us just to see it. I mean, we might already have all this information sorted, but we might've forgotten something or there might be a little prompt that we need.” C07

Likewise, healthcare professionals felt it was crucial for consumers to understand their own individual risk factors associated with opioid prescriptions. In contrast to consumers, professionals felt any resource must go beyond the usual risk factors such as dose and type of medication, to include increased knowledge around how risk could change over time, and around personalized risk factors such as changes in context.

“People could have been on a stable dose for a long time, but other things have changed, and that's where they're not understanding the risks.” P02

Professionals reported experiences of consumers not wanting to engage in conversations about risk, while consumers highlighted how risk-based language could be off-putting. So, both consumers and professionals highlighted the importance of using supportive language to speak about risk factors to not alarm consumers who have been prescribed opioids for acute pain, or to discourage longer-term users with judgmental language.

“I thought let's not start off with the deaths right up front because you're going to scare the bejesus out of everybody. Maybe we'll go with the short-term harms, like constipation and everything” P04

“I think we have to be careful that we are balancing that we're telling people that it is okay to take something if they need it as part of a greater pain management strategy and everything else, but just making sure that we're not sending them the message that we're trying to talk them out of taking an opioid because sometimes it is appropriate” P10

#### 3.1.2 Social/professional role and identity

##### 3.1.2.1 Healthcare professionals role and responsibilities

Consumers felt it was the responsibility of healthcare professionals to educate consumers about opioids and the need for naloxone in the home. Some consumers felt the discussion should start with the prescriber (often their general practitioner), while others felt it could start with their pharmacist. Some consumers were concerned that pharmacists were too busy to have the conversation. A potential mitigation strategy was explored and most agreed that discussions should happen multiple times with different professionals and could also include allied health professionals (e.g., physiotherapists) to ensure the consumer was guaranteed to have the conversation at least once, and ideally with different healthcare professionals.

“Your doctor should have told you about naloxone. [To say]‘Go and talk to your pharmacist’” It just leaves any responsibility for overdose prevention out of the responsibility of the hands of prescribers…I think it should say, “Hopefully your doctor would've mentioned naloxone or suggest you get it from your pharmacy, if not, get it from your pharmacy.” C09

“It's really important that we've got to get that message about safety, but that means all of us have to be involved, particularly the healthcare professional.” P03

Consumers and healthcare professionals agreed it would be crucial for healthcare professionals, especially pharmacists, to receive training in how to have conversations around overdose and the importance of naloxone uptake. Related to the below theme of past negative experiences, many expressed a preference for pharmacists to lead the conversation, but to do so in non-stigmatizing ways. Professionals agreed that healthcare professionals should be the cornerstone of opioid safety conversations that cover the risks associated, alternative treatment options, and the need for naloxone in the home with family members who know how to use it. Some consumers and professionals felt opioid risk and naloxone conversations should happen with every opioid prescription. Most consumers and professionals agreed that in an ideal world there would be trained professionals similar to diabetes educators who would provide specific education for consumers. Healthcare professionals also endorsed the need to use non-stigmatizing language that empowered consumers to ask questions about opioid safety, believing this could open the door for consumers to consider reducing their opioid prescription in the future.

“In an ideal world when you're prescribed an opiate for chronic pain, then it would be great if you could see a pain educator who could be a pharmacist trained to just answer questions. You could have family members attend it with you” P10

“Targeting, again, healthcare professionals to treat patients without stigma and without judgment and empowering and enabling the public in somehow getting to say it's okay to ask questions.” P02

##### 3.1.2.2 Experiences of stigma

Almost all consumers recounted negative experiences with healthcare professionals and prescription opioids. Some felt like they had been forced to wait prolonged periods to pick up their prescription as some form of test by the pharmacist. Others had experiences of professionals minimizing their experience of pain (e.g., being told to try mindfulness instead of their prescription) or that they need psychological help. These past negative experiences with professionals resulted in consumers being wary of approaching opioid safety discussions with their professionals for fear of repeated negative experiences.

“They don't understand the importance of it I think, how absolutely critical it is to get those medications… I have felt tested by a pharmacist to see how much I'm going to put up with, how long I'm going to wait, when am I going to come back?” C01

“I had another doctor say, ‘They're doing all this stuff about brain plasticity. So just stop being in pain.'”

“One of the things that I hate doing when I go to the chemist is knowing what to say because there is that level of judgment sometimes” C03

Professionals too had heard examples of consumers facing stigma from other healthcare professionals. All agreed it was the responsibility of healthcare professionals to provide education around opioid safety and naloxone using non-stigmatizing language. Some community pharmacists had experiences of consumers assuming they were being chastised by the pharmacist for bringing up these conversations while other professionals spoke about the need to educate consumers about their right to seek out other professionals if they were facing stigma.

“Some patients when you talk about the locks [a safety behaviour to keep opioids in a locked cabinet], and they immediately say, ‘you're assuming that I'm drug dependent person, a drug user’. And then you've got them really offside” P12

“Or even giving the person the right or it's okay to change your health professional if you're not happy with the way that you are being potentially stigmatized or… with pharmacies, that's certainly one of the things that we hear back” P02

#### 3.1.3 Environment, context and resources

##### 3.1.3.1 A conversational aid

Consumers discussed how an online resource could facilitate their conversations with healthcare professionals. For most, it was crucial to be able to approach conversations around prescription opioid use, naloxone and safe use with their own information to ensure more equal conversations. Some consumers specifically requested scripted examples of how to speak to their healthcare professional about the risks associated with opioids, while others wanted examples of how to ask for naloxone in pharmacy settings.

“When you go to speak to your clinician that you feel actually armed with enough information that you can have a discussion with them rather than feeling like you don't know anything and they know everything” C10

The ROOM Tool was seen as useful for supporting conversations, especially as mandated 12-month review and second opinion are now required to enable ongoing opioid prescribing by their general practitioner. Some consumers felt the ROOM Tool would give credibility to their signs and symptoms associated with their opioid use. A minority were concerned that the use of numbers (i.e., using numbers to depict their pain on a scale) to measure pain didn't adequately capture the nuance of their experience, while others felt it would provide a starting point to explore their pain experience.

“Rather than just you saying, ‘I'm in a lot of pain,’ if you can say, ‘Look, I filled this in and this is something…that clinicians have developed,’ it might have more credibility.” C04

“C07: Well, it's just people who've been using opioids for a while, and maybe people who are thinking of tapering because if they're already thinking of tapering, and then they take that questionnaire, that maybe helps them with some ways to verbalize that to their GP maybe C01: Or a way for them to assess how the opioids are affecting them.”

##### 3.1.3.2 Material resources and data ownership

Some professionals were particularly concerned with data ownership indicating the importance of consumers having ultimate control of any information they enter into the Toolkit. They also felt that a living document that tracked their medication could promote opioid safety behaviours. Consumers were less concerned by data ownership, and hopeful that if they were to present their data via a Toolkit to a healthcare professional it would be a credible source and “believed”.

“It's a living document that the consumer has ownership of. So if the consumer's aware of what they can do, and they keep on getting their chronic pain meds because they're knowing they're using them safely. So that's an incentive for them.” P08

#### 3.1.4 Social support

##### 3.1.4.1 Others need to know how to use naloxone

Consumers and professionals agreed that any resource promoting the uptake of naloxone must include instructions for household members on how to access and use naloxone, and engage in related behaviours such as calling the ambulance and putting the person in the recovery position. Consumers and professionals alike felt these behaviours were likely unknown and needed to be explicitly provided.

“I guess the other thing that goes with that is that whatever material we produce hopefully would be useful for families well, to read it, and to understand, because it's pretty hard to do naloxone education if you don't involve family” P12

#### 3.1.5 Beliefs about capabilities

##### 3.1.5.1 Consumers are experts in their own experience

A major barrier to engaging in opioid safety behaviours for long-term consumers was their belief that they were immune to the risks associated with taking prescription opioids. Consumers with new prescriptions, older people, and people who use illicit opioids were viewed as more likely to lack opioid safety behaviour knowledge and therefore at the greatest risk. Conversely, almost all long-term consumers were confident in their own knowledge of their condition and medications, often describing how their own understanding exceeded that of their treating physician or other healthcare professionals, and not perceiving themselves to be at risk.

“Some of us are on pharmaceutical doses that are quite low because we need this to manage our daily life and we're not escalating. We may be slightly dependent, but we're not at risk of overdosing. It's the opioid naive people in my humble opinion, who are at risk and those who engage in risky behaviours such as your illicit users or the elderly.” C02

#### 3.1.6 Inductive analysis—design preferences

Design preferences were explored throughout each workshop and analysis grouped into four key areas—visuals, language, target audience, and information (Table 2). First, the visual preferences included: (i) the use of vector-based illustrations and animations (as opposed to photography), (ii) the use of digital, animated, and print-based media, (iii) a preference for a positive, optimistic and motivating online digital space where users felt safe, and (iv) an easy to navigate and interact website with limited cognitive load. As one consumer explained, design preferences were intrinsically linked to their experience of pain with the potential for design decisions to influence their ability to process the information.

**Table 2:** Design preferences and illustrative quotes.

Design preference theme	Illustrative quote
Visual preferences	“I've done a few online pain course tools and they had photos of real people…I can get a pain flare if I see somebody doing certain things, moving their legs in a certain way, someone that's in pain, so I'd be better with animation.” C10
Language preferences	“A lot of this should be objective language that's encouraging you to learn about the condition, not telling you to be afraid or fearful of it.” C02
Target audience preferences	“it's [example of opioid safety material] trying to do a one fits all approach and I think that has huge risks for misinformation and mistreatment and also the risk of stigmatizing people
Information preferences	“Setting the expectations of what they can expect, and I think that's what opioid users or people need when they're trying to access information. They know exactly where to click and what they're going to get.” C08

Second, the preferences related to language included: (i) using non-targeting/non-stigmatizing language, (ii) avoiding too much text, (iii) avoiding overly clinical language, (iv) using simple and easy-to-understand language, and (v) correctly using and defining common terms and acronyms. Briefly, language and messaging was designed to be non-stigmatizing by validating participants perspectives that for some people opioids are important part of pain management (e.g., “For some people, opioids are the best option for pain relief”), and ensuring existing stigmatizing language used in resources were not included (i.e., listing harms before benefits of opioids, NPS MedicineWise) (59).

Third, preferences related to the target audience included: (i) explicitly defining the target user, (ii) screening individuals to identify their level of risk, and (iii) giving guidance based on their level of risk. Finally, information preferences included: (i) catering to a wide range of audiences from different cultural backgrounds, (ii) giving tailored information, (iii) providing evidence-based, factual information, (iv) providing a clear call to action or next step, and (v) being relevant to the Australian context.

### 3.2 User testing

User testing was conducted with 15 consumers with lived experience of being prescribed opioids for pain relief including 12 long- (more than three months), 1 medium- (2 weeks to three months), and 2 short-term (less than 2 weeks) (Table 1; Supplementary Table S3). User testing feedback provided an additional opportunity to refine the Toolkit (Supplementary Figure S3). Feedback related to either information and resources presented (e.g., instructions provided), or specific features of the website (e.g., interactivity). This section details the positive and negative feedback elicited through user testing (Table 3), quantitative user testing (Table 4) and modifications made a result (Table 5).

**Table 3:** Example positive and negative feedback from end users about prototype v2.

Summary of feedback	Example feedback
Positive feedback	
Information and resources: information is easy to understand	“So this language, learn about the risks and benefits… feels very supportive and encouraging.” C02 “I honestly didn't know anything about this, so I'm actually learning quite a lot and I'm liking that it's for the first time me seeing this. I like that I can fully understand it as well.” CP10
Information and resources: information is important and relevant to me	**“**I like this part here, how it says crafted by consumers and health professionals and it's evidence-based.” P10 “It's tailoring something to my needs so it feels more like it's going to give me the answers that I need.” C03
Information and resources: I will share this information with others	**“**It's good to have a safety plan and to share it with someone I'm living with because if I was to take an overdose and they don't know that I'm taking opioids, then they wouldn't be able to help me quickly.” C03 “We can make a mistake or take too much or whatever and we need to be aware that that could happen and what to do. And I think it's important to all alert all the people in the family around us.” C06 “I would actually inform my GP that I see every 28 days because they have a huge lack of resources. And to have another resource that they can say basically, I'm too busy here, go and look at this on the internet. Sort yourself out. I think this is a terrific tool that I can tell my doctor about.” C08
Information and resources: resources are useful to keep for later	**“**Wow, that's fantastic. I've got something that I can now save to my desktop.” C04 “I would print off this, I just prefer to have hard copies of things and I would sit down with my boyfriend, so I'd sit down and show him that what to do, especially how to administer the nasal spray if needed.” C10
Website features: interactivity	“I also really liked the questions because they were simple and there were options to tick. It wasn't like I had to write in a lot of things.” C03 “It's really interactive. It's not a boring website or anything. It makes you want to do it and at the same time you're learning so much” C10
Website features: navigation	**“**I think this, for me, this is really nice because I don't have to do it myself. This Toolkit already pointed me to this and I would go on and explore all these different section.” C03 “These are very nicely created pictures or questions and it's very simple and straightforward to understand what it is trying to tell me. So well done there.” C11
Website features: visual design	**“**This design is easy, it's not too fussy. I like the use of the animated drawings as opposed to having photos. There's something for everyone in terms of static images, even if you did nothing except watch the video.” C02 “I like it because it's concise and again, I really like that yellow colour, I like the bound boxes. It's very easy to navigate.” C08 “I like the dot points as well. It doesn't feel too overwhelming.” C10
Negative feedback	
Information and resources: information or instructions are unclear	**“**I'm not clear here what you're asking me. Understanding your risks can support safe use. Oh, so it's looking at what are the risks? Yes.” C01 “The opioid safety Toolkit includes advice on developing a safety plan and using the ROOM tool. What is that?” C15
Information and resources: too much information	**“**I'll be coming back tonight. Kind of had enough information at the moment. I found it's really good. I trust it. It's a good resource. I'd probably be coming back at a later stage. I don't spend hours on websites, so it's all there.” C04
Information and resources: uncertainty about factors external to the website	**“**My concern would be that a lot of doctors wouldn't have time to go through this with the patient.” C01 “Maybe a lot of people wouldn't ask and naloxone because they wouldn't want the pharmacist to think they're abusing their medication.” C01
Website features: navigation is confusing	**“**This is where I'm getting confused. So I would go, okay, we're creating a safety plan. You've asked me and now am I learning about the risk or creating safety plan?” C04 “Only thing because I'm not sure about is because we've got, let's get started up here and then I see about the safety Toolkit underneath and then it says use ROOM tool. So not sure which one I should go first.” C05

**Table 4:** Quantitative metrics collected during user-testing of prototype v2.

Usability issues during user testing	Number of participants who encountered the issue
Experienced issues with user navigation and needed assistance from the researcher	11
Encountered a “bug” (design defect)	7
Task completion rate (able to create a safety plan)	15

**Table 5:** Key areas to improve opioid safety toolkit and summary of modifications made.

Key area for change	Modification made
Information and resources	• Including additional copy to better explain what consumers can expect to see next (i.e., to prime consumers through sign posting) • Including additional copy to explain what the ROOM tool is earlier in the user navigation • Updating navigation menu button names to be clearer and more precise
Website features	• Making minor changes to the user flow to better tailor information to the needs of users (including bug fixes) • Including additional navigational buttons • Adding a visible navigation menu on all pages of the Toolkit to facilitate ongoing navigation • Including a progress bar within the safety plan and ROOM tool to signpost place

#### 3.2.1 Information and resources

Participants in the user testing interviews were generally positive about the Toolkit's information and resources, with most finding the information easy to understand, tailored, relevant and important non-stigmatizing and encouraging in tone. A major positive aspect of the Toolkit identified by participants was the ability to download and keep the resources created (their completed opioid safety plan and their personal answers to the ROOM tool). Most participants spoke about their intention to download the safety plan and share details of emergency naloxone use with their family and/or friends. Others shared that they planned to download and take the ROOM tool to their next doctor's appointment as a conversational guide. In terms of the information presented, some consumers found the website to have too much information to take in in a single session. While other consumers found aspects of the information and instructions unclear thus being usure of what they were being asked to do. In response, modifications were made to the copy on the website. Labels on navigation buttons were updated for clarity. Signposting copy was also added to prime consumers on what they can expect to see next. While additional copy was added to better explain the ROOM tool and its purpose.

#### 3.2.2 Website features

Think-aloud activity in the user testing uncovered a number of positive and negative aspects of the website's user experience and interactivity. Overall, participants appreciated the use of bright colors and icons throughout the design with the layout enhancing information provision. Positively, many participants found the interactivity aspects interesting and simple to use. Similarly, some found the navigation overall simple to follow throughout most of the website with users being funneled to specific tailored information based on their inputs. Others found this funneling confusing or annoying, indicating they expected to be directed to different information based on their inputs. In response, changes were made to the user flow to better tailor information to the needs of consumers, including adding additional navigation buttons, a menu visible on all pages of the Toolkit, and a progress bar to indicate users' progression throughout the creation of their safety plan and the ROOM tool.

Finally, a dissemination plan was created to ensure pharmacists across Australia are prepared for consumers to request naloxone, while consumer specific social media content was developed to increase awareness of the Toolkit among consumers.

### 3.3 The opioid safety toolkit

**Figure 2:**
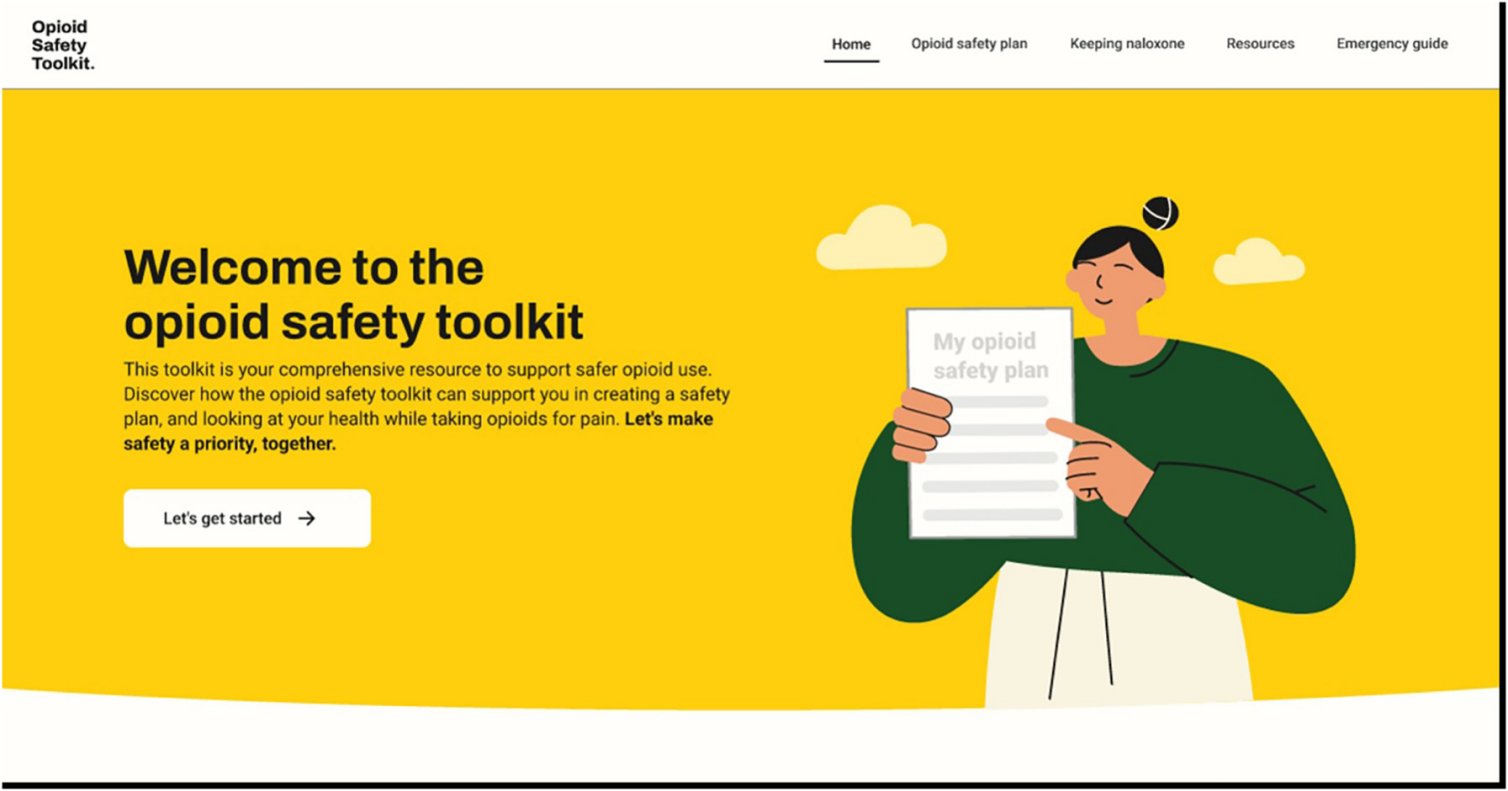
Opioid safety toolkit webpage “Screenshot from:Opiod Safety Toolkit, https://saferopioiduse.com.au/”.

The final web application (60) (Figure 2) includes different user flows to direct consumers to different experiences based on their needs, updated versions of the previously co-designed resources (ROOM tool, consumer-facing leaflet and videos), newly co-designed resources to increase safety behaviours, and a naloxone pharmacy finder tool. Specific Behaviour Change Techniques are included in the tool including information about health consequences, instruction on how to perform the behaviour, demonstration of the behaviour, credible source, social support (practical), and restructuring the physical environment (see Table 6 for operationalisation).

**Table 6:** Behaviour change techniques employed in the intervention component or content of the opioid safety toolkit.

Behaviour change technique (50) and description	Intervention component/content
Information about health consequences “Provide information (e.g., written, verbal, visual) about health consequences of performing the behavior”	The Toolkit presents the potential risks of opioids, for example: “Opioids are medicines taken to help reduce pain. They work on the central nervous system to slow down messages (nerve signals) between the brain and the body. Like all medicines, opioids can also produce side effects. These can range from constipation to more severe side effects like slowed heart rate or breathing”
Instruction on how to perform behaviour “Advise or agree on how to perform the behaviour”	Instruction are provided on how to recognize the symptoms of opioid overdose and provide naloxone, for example: “To administer naloxone as a nasal spray, spray one dose into the nostril. If using the injection, inject one dose into the outer shoulder or thigh muscle. Note the time you use the nasal spray or injection. If there is no response after 2–3 min, repeat the dose. The effects of naloxone are temporary—they last for approximately 30–90 min. Ensure that emergency services are on their way. Call emergency services on 000”
Demonstration of the behaviour “Provide an observable sample of the performance of the behaviour, directly in person or indirectly e.g., via film, pictures, for the person to aspire to or imitate”	The safety plan section of the Toolkit provides video and image depictions of how to administer naloxone and place someone in the recovery position
Credible source “Present verbal or visual communication from a credible source in favour of or against the behaviour”	The Toolkit highlights that the Toolkit was created in collaboration with consumers, healthcare professionals, and advocacy groups
Social support (practical) “Advise on, arrange, or provide practical help (e.g., from friends, relatives, colleagues, ‘buddies’ or staff) for performance of the behaviour”	The Toolkit encourages user to share their safety plan with others in their home, for example: “It is best to share your safety plan with the other people in your home so that, in the event of an emergency, they will know what symptoms to look for and what to do”
Restructuring the physical environment “Change, or advise to change the physical environment in order to facilitate performance of the wanted behaviour or create barriers to the unwanted behaviour (other than prompts/cues, rewards and punishments)”	The Toolkit recommends users download or print their safety plan and ROOM tool for easy access and to share with their friends or family (safety plan) healthcare professional (ROOM tool). The Toolkit advises users to obtain naloxone from their pharmacy, there is a “pharmacy finder” tool that allows users to find nearby pharmacies that currently stock naloxone

## 4 Discussion

This study aimed to design and develop a digital Opioid Safety Toolkit for people who are prescribed opioids for pain. A secondary aim was to extend the integration of implementation science and HCI by embedding a well-known behavioural and implementation science framework, the TDF, with the Double Diamond design process (40, 45, 46). Through participatory co-design approaches underpinned by the TDF, nuanced barriers and facilitators to engaging in opioid safety behaviours (i) making a safety plan, (ii) speaking with their healthcare professional about opioid safety, and (iii) having naloxone in the home and ensuring others know how to use it were identified and embedded within the final Toolkit. This research addresses gaps in literature by designing digitally mediated naloxone interventions for people who have been prescribed opioids for pain, rather than for people who use illicit opioids or misuse opioids, including their risk reduction perspectives (25). Furthermore, this extends previous community pharmacy naloxone intervention research that has predominantly been based in the United States (61). Themes relating to the barriers, facilitators and design of the digital opioid safety Toolkit emerged from workshops with consumers and experts. Using the TDF as a lens to interpret the deductively coded data, dominant themes included Knowledge; Social/Professional Role and Identity; Environment, Context and Resources; Social Support; and Beliefs about Capabilities. Inductive themes within each domain related to the need for increased public awareness and understanding personalised risk (Knowledge), healthcare professionals' role and experiences of stigma (Social/Professional Role and Identity), the need for a conversational aid and material resources and data ownership (Environment, Context and Resources), the need for others to know how to use naloxone (Social Support), and consumers' expertise in their own lives (Beliefs about Capabilities).

Past experiences of stigma were discussed by all consumers and most experts as one of the main barriers to consumers engaging in opioid safety behaviours. Research on stigma and OUD suggests that stigma is experienced in complex ways, conceptualised through an interplay of individual, social, and societal levels (62, 63). For instance, stigma has been shown to influence the way doctors interact with patients who are using prescription opioids (64, 65) and can decrease the likelihood of requesting naloxone from a pharmacist (66). Design considerations had a great effect on consumers’ perception of stigma across workshops. This finding was related to the consumers' belief about their own capabilities, with long-term consumers believing that they were immune to opioid risks and therefore not in need of any additional information. Consumers' propensity to underestimate their personal opioid overdose risk are supported by previous research wherein patients with non-cancer pain did not consider themselves at risk of overdose (67–69). This perception of risk also aligns with the general populations propensity to see their own risk for negative health consequences as low (70, 71).

To address these barriers, the Toolkit needed to include language that would validate the experiences of people prescribed long-term prescription opioids. For example, positive framing of opioids (that acknowledges pain relief properties), rather than risk-based framing, was seen to be more acceptable and less stigmatising. This is in direct opposition to currently available Australian opioid education resources which tend to rely heavily on risk-based language (59). This finding was integrated into the design through different user flows for consumers based on the length of their prescription. As such, the design results in tailored and personalised information ensuring long-term consumers only access necessary information that leads them to create an opioid safety plan and engage with the ROOM tool. In contrast, people taking opioids in the short term (commencing opioids in the last 2 weeks) receive more information regarding the risks, benefits, alternative treatment options for opioids, and set expectations for a short duration of use. However, as discovered in the user testing the user flow was too prescriptive and as a result confusing for some users, a solution to this was the additional of a navigation bar to allow users to also move freely throughout the Toolkit. Furthermore, the majority of people who are prescribed opioids in Australia are prescribed for non-cancer chronic pain (9). As such, it was important to consider the pain experiences of consumers in the design of the Toolkit. For example, a couple of consumers commented that seeing imagery of real people could exacerbate their pain symptoms. As such the design only included vector-based illustrations as to not inadvertently cause pain to consumers interacting with the Toolkit.

Healthcare professionals' role was seen to both help and hinder opioid safety behaviours. On one hand, consumers were clear in their preference for healthcare professionals to initiate conversations about opioid safety. This is consistent with past research that showed a high level of acceptability by consumers for healthcare professionals providing naloxone (15). The work by Nielsen et al. (15) was conducted to explore healthcare professionals concerns that conversations with consumers would result in offence being caused to consumers. A perception that has persisted, and echoed in both the workshops and user testing interviews. The fear of causing offence may be one of the barriers limiting the distribution of naloxone through the Australia Government's take home naloxone program. Previous research has advocated for the use of multifaceted interventions that address both the demand and supply of naloxone uptake (36). Findings from this work supports the need for multifaceted approaches to address low provision for people prescribed opioids, and are reflected in the dissemination plan for the Toolkit.

In this study, a facilitator for opioid safety behaviours was a conversational aid to support consumers to know how to speak to their healthcare professional (supporting the demand side). This finding was triangulated in the user testing interviews with the majority of consumers expressing their intention to use the ROOM tool as a conversational aid with their clinician. Future scalability of the Toolkit relies on buy-in from multiple stakeholder groups, including healthcare professionals, administrators and government policy makers. To ensure scalability, the provision of naloxone and information regarding its importance must become standard practice. To achieve this we also focus on the supply side with a healthcare professional facing campaign (the details of which will be reported elsewhere).

This campaign aims to increase healthcare professionals' awareness of the Toolkit, encourages consumers to obtain naloxone from their pharmacists, and builds on previous work which included developing a language guide for pharmacists on how to initiate conversations with consumers about opioid safety (54). The dissemination plan follows the developed brand guidelines to increase credibility across assets and includes text messages for pharmacies to send on dispensing opioids for a consumer which integrates into their current text message services, print posters, presentations at national pharmacy conferences, advertisements in industry magazines, and a dedicated website for pharmacists with additional resources. The dissemination plan will be evaluated by triangulating qualitative and quantitative metrics across key assets and activities. For example, website analytics for the dedicated pharmacist resource including traffic and resource downloads; the number of pharmacies that send text messages; Electronic Direct Mail open rates; and naloxone distribution supply data. Additional dissemination factors will be evaluated for consumers. For example, patient reach will be evaluated through website traffic to the toolkit as well as SMS open rates.

### 4.1 Strengths and limitations

A key strength of this study is the inclusion of consumers with lived experience of being prescribed opioids for pain relief and experts who are responsible for prescribing or dispensing prescription opioids as well as academics and consumer and professional body advocates. Co-design with this diverse group of stakeholders resulted in nuanced insights across individual, organisational and system level factors influencing safety behaviours. The TDF combined with the Double Diamond provided useful guidance throughout the study. The Double Diamond itself was useful in developing the aims and activities of the workshops (e.g., exploration, synthesis, ideation, testing), while the TDF helped to explore factors affecting opioid safety behaviours from multiple perspectives. This triangulation in data has been useful in ensuring the overall design met the needs of multiple stakeholders. For example, themes around stigma, health professional role and beliefs about capabilities were better understood by exploring the ways in which barriers and facilitators to behaviour impact both consumer and healthcare professionals' behaviour. Furthermore, the use of the TDF as a lens for analysis aided the selection of evidence-based behaviour change techniques (Table 4), that mean the intervention itself grounded in behaviour change theory.

Finally, limitations to this study should be noted. The consumer participants (13 in the workshops and 15 in interviews) included in this study are unlikely to reflect the broad and diverse experiences of Australians who are prescribed opioids for pain relief. Although attempts were made to include participants from diverse jurisdictions, majority were from the east coast of Australia and are likely to have an interest in the topic area. Similarly, the expert participants in this study are likely to introduce some selection bias in that those who participated are more likely to be interested in prescription opioid safety. Additional research has included a broader sample so findings relating to effectiveness are generalizable. This includes those who are less interested in or do not have expertise in the subject area through a randomized controlled trial and participant qualitative interviews of their experience with the Toolkit (the results of which will be published elsewhere). Furthermore, as this study included co-design and user testing, it is not possible to discuss the Toolkit's effect on the safety behaviours themselves. These results will be published elsewhere.

## 5 Conclusion

Through a co-design process with consumers and professionals, a digital Opioid Safety Toolkit was designed that can help consumers identify their own opioid risk factors and monitor their outcomes with opioids over time. The digital Toolkit is currently being disseminated in pharmacies nationally in Australia, following a randomised controlled trial confirming its efficacy and acceptability. The co-design process adapted a novel approach to designing with implementation in mind by combining the Double Diamond with the TDF (40, 45, 46). Results from the co-design activities indicate multiple intersecting themes influencing behaviour change techniques and design elements embedded throughout the Toolkit. Translation of previously co-designed evidence-based tools into a web-based Toolkit has the potential to increase the reach, uptake and impact of safety behaviours to empower and support consumers with lived experience of being prescribed opioids.

## Data Availability

The raw data supporting the conclusions of this article will be made available by the authors, without undue reservation.
