# Supplementary material for: An evidence-based digital prescription opioid safety toolkit for national dissemination: co-design and user testing
Source: Front Digit Health. 2025 Jul 11;7:1600836. doi: 10.3389/fdgth.2025.1600836 (PMC12291169; doi:10.3389/fdgth.2025.1600836)

Supplementary Material

1 Supplementary materials include:

1. Demographics of consumers in workshops and which workshops they attended
2. Demographics of professionals in workshops and which workshops they attended
3. Demographics of consumer participants in user testing interviews
4. Journey Mapping Activity Completed by Consumers in Workshop One
5. Example of Prototype v1
6. Example of Prototype v2

1. Supplementary Table 1: Demographics of consumers in workshops.

**Participant**	**Gender**	**Duration of prescription opioid**	**Research Activity**		
			Workshop 1	Workshop 2	Workshop 3
P01	F	Long term prescription	X	X	
P02	M	Long term prescription	X	X	X
P03	F	Long term prescription	X		X
P04	F	Long term prescription	X	X	
P05	F	Long term prescription	X		
P06	F	Long term prescription	X		X
P07	F	Long term prescription	X	X	
P08	F	Long term prescription	X		X
P09	F	Long term prescription	X		
P10	F	Long term prescription	X	X	X
P11	F	Short term prescription, carer			X*
P12	M	Short term prescription			X*
P13	F	Short term prescription			X*

2. Supplementary Table 2: Demographics of Healthcare Professionals and Stakeholders.

**Participant**	**Gender**	**Expertise**	**Research Activity**		
			**Workshop 1**	**Workshop 2**	**Workshop 3**
E01	F	Academic Pharmacist (opioid deprescribing specialist)	X	X	
E02	F	Pharmacist in GP practice	X	X	X
E03	F	Stakeholder advocate	X	X	

E04	F	Academic Pharmacist	X	X	X
E05	F	Pharmacist (Pain management specialist)	X		
E06	F	Pharmacist in GP practice	X		
E07	M	Pharmacist (alcohol and other drug treatment focussed)	X		
E08	F	Community pharmacist	X		
E09	F	Community pharmacist	X	X	X
E10	F	Community pharmacist	X		
E11	F	Pharmacist (Community setting)	X	X	
E12	F	Prescriber/Pain Specialist	X	X	X
E13	M	Stakeholder advocate	X		

3. Supplementary Table 3: Demographics of consumer participants in user testing interviews.

**Participant Number**	**Gender**	**Opioid Prescription Length**	**Operating System**	**Browser**	**Location (Metro/Regional, State)**
P01	F	More than 3 months	Mac	Chrome	Regional, NSW
P02	F	Less than 2 weeks	PC	Edge	Metro, VIC
P03	F	2 weeks to 3 months	Mac	Safari	Metro, VIC
P04	M	More than 3 months	Mac	Safari	Metro, QLD
P05	F	More than 3 months	PC	Edge	Metro, VIC
P06	F	More than 3 months	Mac	Firefox	Metro, SA
P07	F	More than 3 months	Mac	Safari	Metro, NSW
P08	M	More than 3 months	Android	Samsung	Metro, SA
P09	F	Less than 2 weeks	PC	Chrome	Metro, VIC
P10	F	More than 3 months	Mac	Safari	Regional, VIC
P11	M	More than 3 months	Mac	Chrome	Regional, NSW
P12	M	More than 3 months	Mac	Safari	ACT
P13	F	More than 3 months	Mac	Chrome	Regional, NSW
P14	F	More than 3 months	PC	Edge	Regional, NSW
P15	M	Less than 2 weeks	iPad	Safari	Regional, NSW

4. Supplementary Figure 1: Journey Mapping Activity Completed by Consumers in Workshop One.

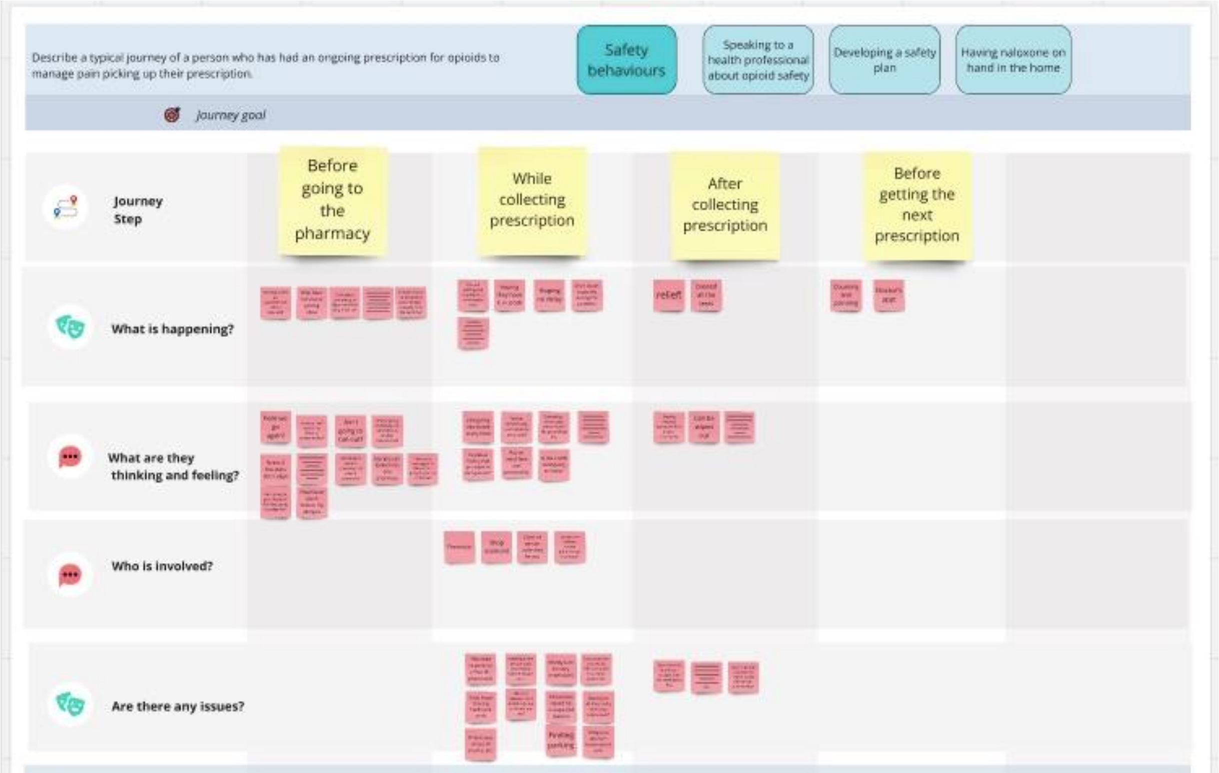

5. Supplementary Figure 2: Example of Prototype v1.

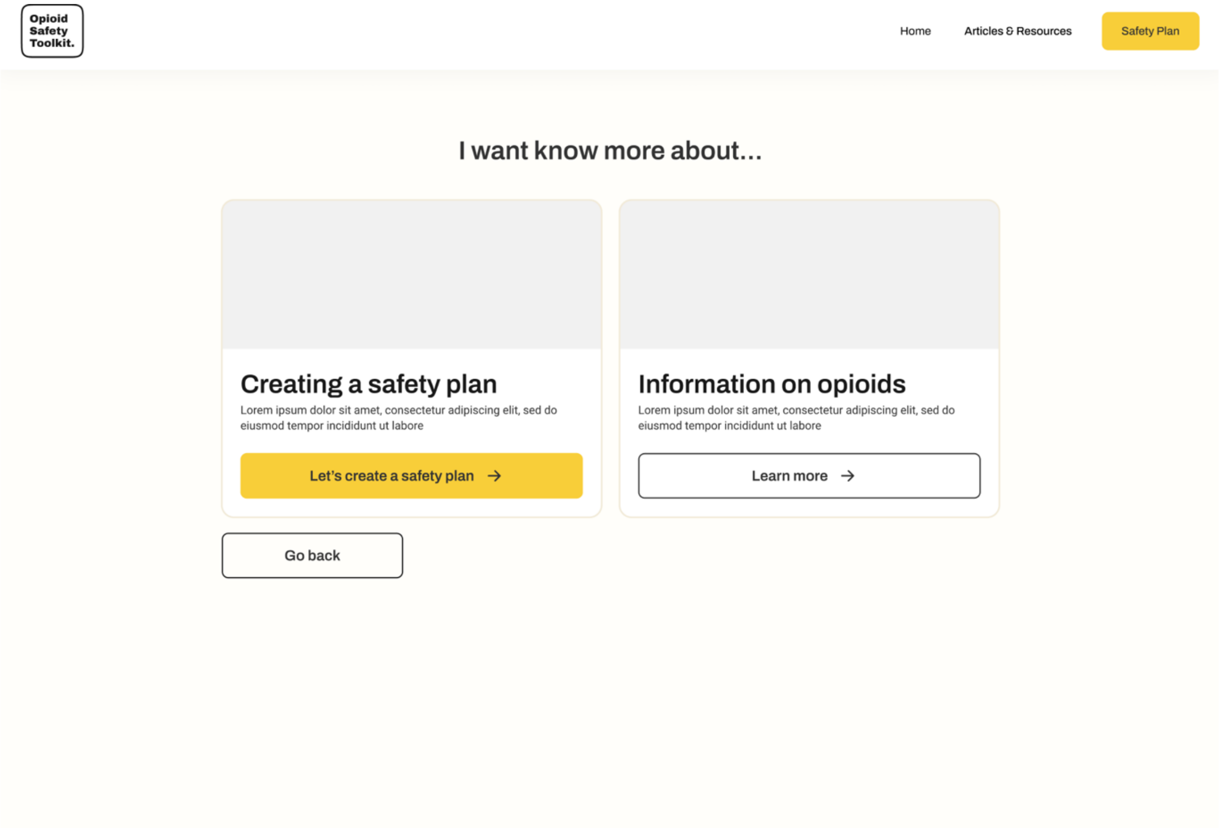

6. Supplementary Figure 3: Example of Prototype v2.

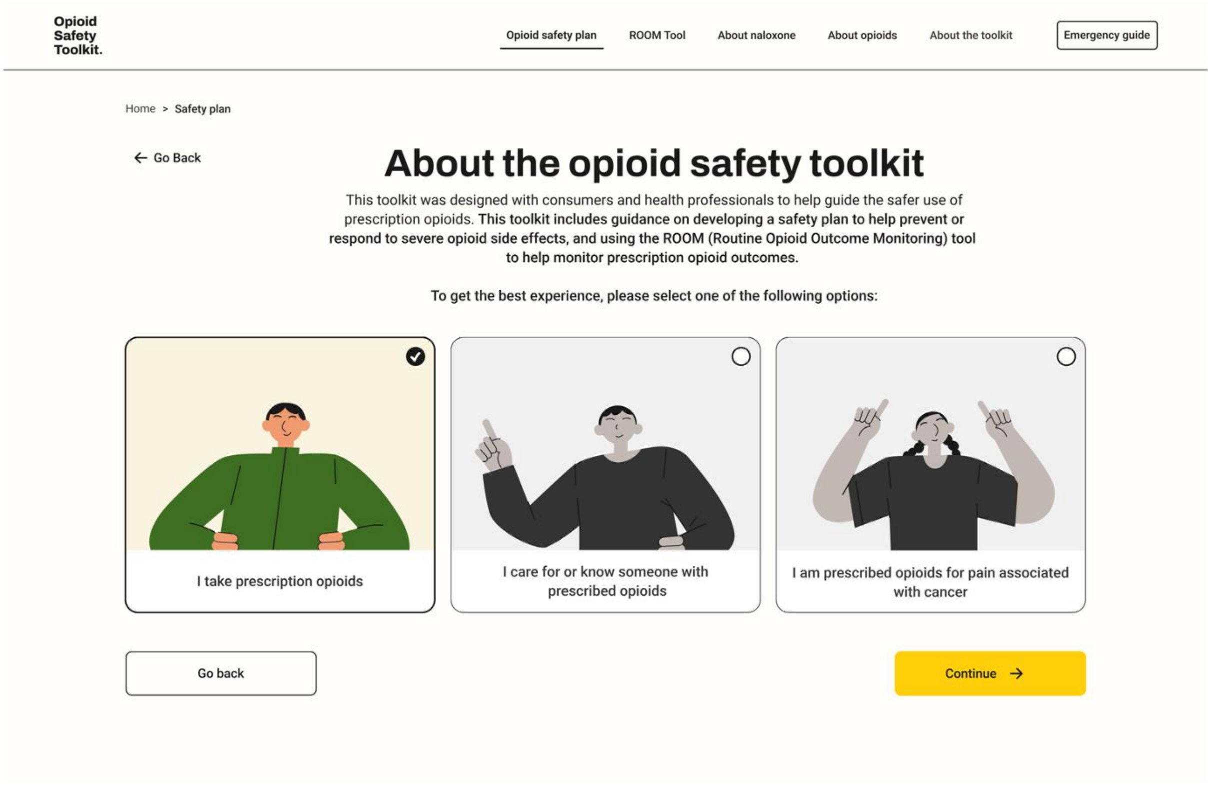

Supplement: Supplementary file 1 [file Datasheet1.pdf]
